# Influence of a Reclaimed Sand Addition to Moulding Sand with Furan Resin on Its Impact on the Environment

**DOI:** 10.1007/s11270-015-2707-9

**Published:** 2015-12-06

**Authors:** Mariusz Holtzer, Rafał Dańko, Angelika Kmita

**Affiliations:** AGH University of Science and Technology. Faculty of Foundry Engineering, Reymonta 23 street, 30-059 Krakow, Poland; AGH University of Science and Technology. Academic Centre for Materials and Nanotechnology, al. A. Mickiewicza 30, 30-059 Krakow, Poland

**Keywords:** Foundry sands, Air pollutions, Leaching, Emission, PAHs, BTEX

## Abstract

Metalcasting involves having a molten metal poured in a hollow mould to produce metal objects. These moulds are generally made of sand and are chemically bonded, clay-bonded, or even unbounded. There are many binder systems used. Binders based on furfuryl resins constitute currently the highest fraction in the binders no-bake group. Moulding sand, after knocking out the cast, is partially reclaimed, and the remaining part, known as waste foundry sand is used or stored outside the foundry. In this case, the environment hazardous organic compounds and metals can be leached from the moulding sand, thus causing pollution of water and soil. Also during the casting moulds with molten metal, they emit pyrolysis gases containing many different compounds, often dangerous from the BTEX and PAH group, which has adverse impacts on the environment and workers. The article presents the results of research on the impact of the regenerate addition to the moulding sand matrix on emitted gases and the degree of threat to the environment due to leaching of hazardous components. Therefore, for the total assessment of the moulding sands harmfulness, it is necessary to perform investigations concerning the dangerous substances elution into the environment during their management and storage, as well as investigations concerning emissions of hazardous substances (especially from the BTEX and PAHs group) during moulds pouring, cooling, and casting knocking out. Both kinds of investigations indicated that reclaimed sand additions to moulding sands have significantly negative influence on the environment and working conditions.

## Introduction

Metalcasting involves having a molten metal poured in a hollow mould to produce metal objects. The casting is then left for a certain time until it cools down. After cooling, the casting is removed from the mould. The sand mould are used one time and destroyed after pouring. These moulds are generally made of sand, and are chemically bonded, clay-bonded, or even unbounded. In the sand moulding processes, there are many binder systems used (organic and inorganic), but most of them are variations on a few basic chemicals. Some of the chemical compounds that are used are phenolic, urethane, and furan resins. Binders based on furfuryl resins constitute currently the highest fraction in the no-bake binders group. Furan resins are commonly used for moulding and core making of medium and large size parts, for small and medium bath production and for all alloy types. Furan binders provide excellent mould and core strength, cure rapidly, and allow the sand with which they are used to be reclaimed at fairly high yields (Holtzer et al. 2015).

In foundry industry, millions of tones of spent materials are disposed in the world. Over 70 % of the total amount of the dumped waste materials consists of sands. Admittedly, in several countries, reclamations of waste foundry and core sands became a standard, but the application 100 % of a reclaimed sand as a matrix is rarely possible. A waste sand reclamation process allows recovering a sand matrix. However, in several foundry plants, in spite of having the reclamation installations, some amounts of moulding sands after a casting knocking out must be removed from a foundry plant and either managed outside it, or—in the last resort—stored. Most often this is moulding sand which was prepared already with a reclaimed sand fraction. Not all foundry plants have reclamation lines. It is assumed that, on average, 1 Mg of castings generates 0.8–1.0 Mg of waste foundry sands. With the world production of 30 million Mg (in 2013) of iron castings made with chemically bonded moulding sands and with the reclamation rate of about 40–50 %, we can assume that each year about 15–18 million Mg of used mould sand must be managed outside the foundry or stored (Modern Casting 2014).

According to the provisions in force, the storage of waste foundry sands (WFS) should be treated as the last resort. The reasons are as follows: high storage costs, irretrievable losses of sands as a raw material, remediation problems, and regulation restrictions (Deng and Tiskalsky 2008). Many of the waste moulding sands, especially green sands from ferrous and aluminum foundries, are useful as a substitute for the virgin sands. Therefore, various ways of the management of waste foundry sands are looked for: speedway embankments, terrain leveling, building of rolled fills, etc.

Beneficial reuses of waste foundry sand span a variety of applications related to infrastructure engineering and rehabilitation works, e.g., highway embankment construction (Ham et al. 1990; Kleven et al. 2000) ground improvement, and concrete (Naik et al. 2001) hydraulic barrier or liner. These alternate applications offer cost savings for both foundries and user industries, and an environmental benefit at the local and national level. For many years, the waste sands generated by foundry industry were successfully used as landfill materials (Ham et al. 1986; Kniese 1992). But disposal by landfill of waste sands is becoming an increasing problem as legislation is getting tighter and economic as disposal cost by current practices increases rapidly (Stevenson 1996). In last 10 years, an increasing attention has been put on the chemistry, leaching characteristics, and alternative application of waste foundry sands (Baker 1997; Echard et al. 1995; Ham et al. 1981; Leidel et al. 1994; Stanforth et al. 1988; Alves et al. 2014). However, because trace metals may accumulate in the moulding sands during casting there is concern over the contamination of terrestrial and aquatic environments (Dungan and Dees 2009). During casting processes, in moulding sands with organic binders and green sand, under an influence of high temperatures of liquid metals, the pyrolysis of substances occurs and—as a result—gases, often harmful, are generated (Allen et al. 1991; Liang and Tsay 2010). The harmful gases affect the health of workmen and nearby residents, and cause public protests. Among the 189 HAPs listed in the 1990 Clean Air Act Amendment (CAAA), some 40 compounds have been identified in the air emissions from the foundries; and over 90 % of the foundry HAPs are organics (Fox et al. 2002; Wang and Cannon 2007). These HAPs are released during metal pouring, mould cooling, and casting shakeout when the carbonaceous additives are exposed to the casting heat. As numerous investigations indicate, including the authors own (Holtzer et al. 2014; Holtzer et al. 2013) the formed substances, out of which several are dangerous, e.g., BTEX and PAHs, are partially condensed on matrix grains and together with moulding sands are undergoing the mechanical reclamation process (Humfrey et al. 1996; Ribeiro and Filho 2006; Fox et al. 2002).

During this process, a certain amount of these substances is removed together with after reclamation dusts (which causes that these dusts become hazardous wastes) but a significant amount remains (depending on the reclamation process effectiveness) and together with a reclaimed sand enters into the composition of the new moulding sand prepared with its fraction. These substances, on one hand, will again form gaseous phases under the influence of high temperatures and will pollute the environment, and on the other hand, together with newly formed—during pouring—substances will become hazardous for the environment since they will be eluted into it (Dungan 2006; Ji et al. 2001).

To be able to estimate possibilities and to select directions of a waste foundry sand management, these wastes must be subjected to several technological and environmental tests. One of them is the elution test, which allows assessing the waste harmfulness when it is subjected to, e.g., atmospheric factors influence (when used for road building or when stored). In case of storing, it is important to determine which type of dumping site is suitable for the given waste since there are various storage costs.

Economical utilization of waste foundry or core sands decreases the amount of wastes transferred into the environment and protects natural resources of the globe. Thus, looking for various ways of wastes management becomes more and more intensive (Deng and Tiskalsky 2008; Holmgren 2010).

Therefore, the estimation of the harmfulness of the given moulding sand should contain emissions during pouring the mould with liquid metal, as well as elution tests of the sand. These tests should encompass moulding sands prepared on a fresh sand matrix and on a matrix with the reclaimed sand fraction (Holtzer et al. (2004a, b)).

A special attention should be directed to the emission of substances from the BTEX and PAHs groups, since a lot of them are carcinogenic and mutagenic. The results of broad investigations of gas emissions for some systems: furfuryl resin—hardener in a temperature range up to 1350 °C was presented in the paper (Liang and Tsay 2010). Concentrations of the following substances were determined as follows: BTEX, thiophene, CO, SO_2,_ and CS_2_. In addition, the authors found the presence of hydrogen in heated sands, which indicated that some PAHs would be formed in the casting process, and that they were easy to accumulate within the moulding sand matrix or to disperse in the air. Studies concerning new catalysts, which generate smaller amounts of these dangerous substances, are under way (Zhang et al. 2014; Renhe et al. 2011; Yuyan and Yingmin 2009).

Therefore, the aim of the hereby investigations was not only the determination of the emission of substances from the BTEX group but also from the PAHs group from the moulding sands with furfuryl resin under the pilot plant scale, as well as the determination of the elusion degree of dangerous substances in case when waste sands were stored or economically utilized outside the foundry plant. Investigations were carried out for moulding sands prepared both on fresh sands and with a reclaimed sand addition. All these elements form the overall assessment of the harmful influence of the given moulding sand for the environment and for the employees. Due to this, it is possible to protect the nature against hazardous substances.

## Materials and Methods

Moulding sands with a binder based on furan resin were tested. This is urea-furfuryl resin of the free furfuryl alcohol content below 25 %. In addition, it contains the following: 0.1–0.2 % of free formaldehyde, 2.5–3.20 % of nitrogen, <3.0 % of ethyl alcohol, and <2 % ethanediol. Hardener for this resin contains 65 % of p-toluenesulfonic acid and 1 % of sulfuric acid. Matrices of these moulding sands were of high-silica sand and a reclaimed sand (of a lost on ignition, LOI = 0.78 %) obtained by the mechanical reclamation process, and originated from the moulding sand of the same composition as the tested one (a temperature of liquid metal poured into the mould was equal to 1350 °C, metal mass: moulding sand mass = 1:3).

Moulding sands of the following composition were prepared:

- Moulding sand 100PS containing 100 % of a fresh sand, 1 % of resin, and 0.5 % of a hardener, LOI = 1.32 %;
- Moulding sand 50PS50R containing 50 % of a fresh sand and 50 % of a reclaimed sand, 1 % of resin, and 0.5 % of a hardener, LOI = 2.18 %;
- Moulding sand 100R containing 100 % of a reclaimed sand, 1 % of resin, and 0.5 % of a hardener, LOI = 3.15 %.

Cores made of these moulding sands were poured with liquid cast iron of the temperature of 1350 °C, and after the casting knock out, the samples were taken.

### Leach Test

Because there is no relationship between the total metal concentration in a solid waste and its leachability, leaching tests are often used to determine the potential of a solid waste to contaminate ground water.

Elusion tests were made according to the Polish standard PN-EN12457–4:2006, Basic test (Polish Standard: PN-EN 12457–4 2006).

The obtained results were compared with the allowable values of substances eluted from wastes stored in dumping site for neutral wastes (acc. to the Polish standards) (Regulation of the Minister of Economy 2013) and toxicity threshold values of the Toxicity Characteristic Leaching Procedure (TCLP) (US EPA). In the Polish test, deionized water of pH = 6 is the waste leaching liquid at the ratio of waste: water = 1:10, while in the TCLP test the solution of pH = 4.9 is the leaching liquid. Therefore, the leaching results, especially of metals, obtained in the Polish test can be underrated in relation to the results that would be obtained in the TCLP test (US EPA - Environmental Protection Agency, Test Methods for Evaluating Solid Wastes).

### Investigations of the Gases Emission

Investigations of the gases emission in the test foundry plant were performed according to the original method developed in the Faculty of Foundry Engineering, AGH UST (Polish Patent, No P-398 709 2012).

**Fig. 1:**
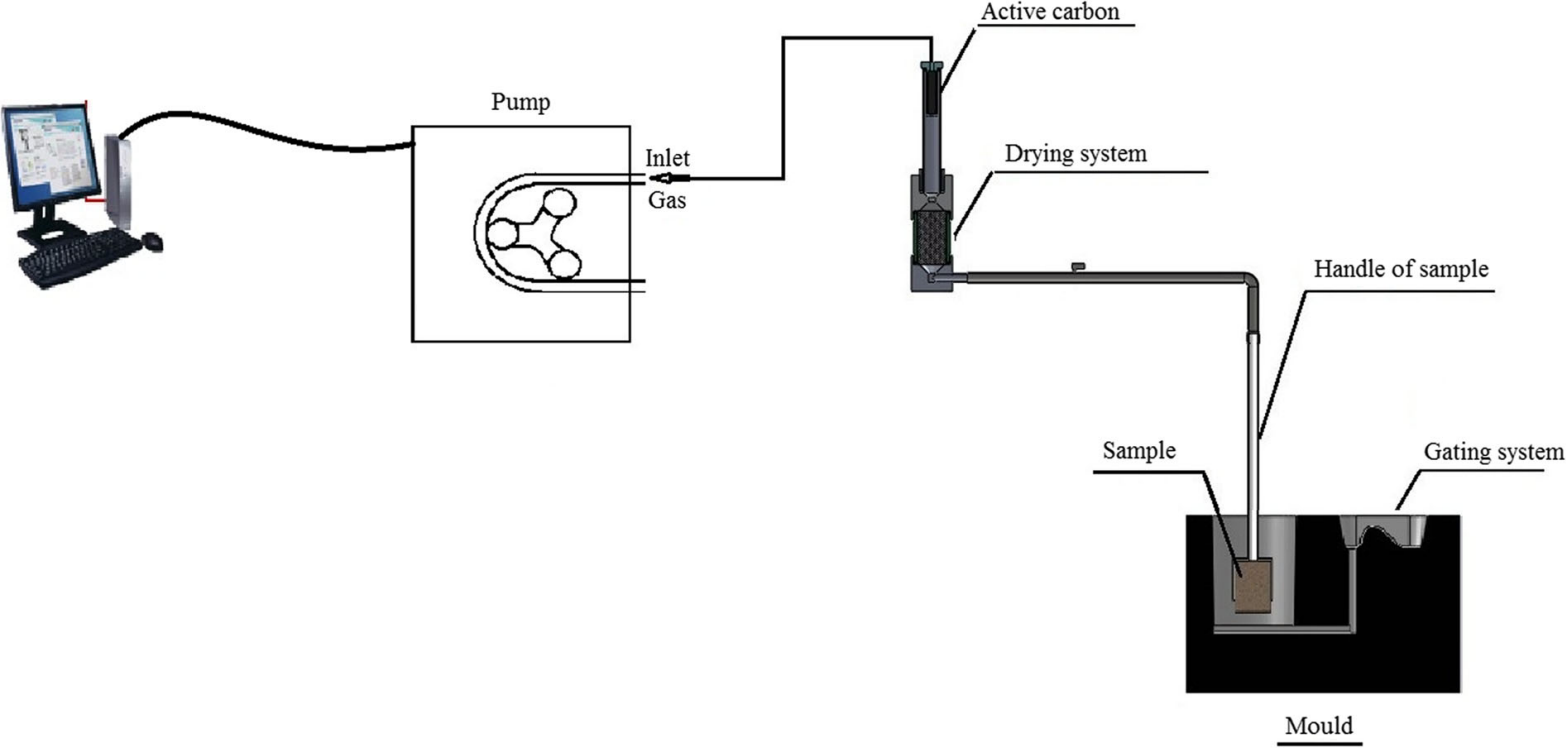
Scheme of stand of measurement of gas volume and BTEX and PAHs emission (Polish Patent, No P-398 709 2012)

The schematic presentation of the experimental stand is given in Fig. 1. A sample of the investigated moulding sand of a roll shape of dimensions Φ 50 × 50 mm, weight about 150 g, compacted by a moulder’s rammer stroke, is poured with liquid cast iron of a temperature of 1350 °C. The liquid metal mass was 9 kg. Gases emitting from the sample—after pouring it with liquid metal are led by means of a steel pipe via the drying system and the capsule with active carbon (during the BTEX measurement) or polyurethane foam^1^ (during the PAHs measurement) into a tightly sealed container with liquid, from which they push out the liquid. The weight of displaced liquid was measured as a function of time. The whole mould (weight 24 kg) is made of green sand.

Two layers of active carbon separated from each other were placed in the glass tube. The first layer (A) containing 700 mg of active carbon constituted the main adsorption place, while the second layer (B) containing 200 mg of active carbon was of a control character, providing information on an eventual ‘breakthrough’ of the first adsorption layer.^2^ The active carbon layer with adsorbed organic substances was extracted in diethyl ether. The analysis was carried out by the gas chromatography method with the application of the flame-ionizing detector (FID) (TRACE GC Ultra THERMO SCIENTIFIC).

At the determination of compounds from the PAHs group, a part of hydrocarbons was condensing on the formed dust particles and together with them was deposited on the filter placed in the capsule before the polyurethane foam. Therefore, at determining the total amount of generated PAHs, the polyurethane foam as well as the dust on the filter were analyzed. Both, the filter and foam were extracted by toluene and then separated from the matrix in hexane. Extracts obtained for individual samples were analyzed by the gas chromatography technique (FOCUS GC) coupled with the mass spectrometry ISQ THERMO SCIENTIFIC (GC/MS).

## Investigation of Results and Their Discussion

### The Elution of the Moulding Sands

The overall results of the elution of the moulding sands on the matrices: fresh sands with the 50 % reclaimed sand addition and of the reclaimed sand only are given in Table 1. The allowable concentrations of substances, which can be eluted from the wastes stored in the given dumping site, are listed in Table 2. Toxicity threshold values of TCLP test (Toxicity Characteristic Leaching Procedure—Regulatory Levels) was given for the comparison.

**Table 1:** The concentrations of leached substances from the moulding sand with furan resin (content of free furfuryl alcohol <25 %) (ds—dry substance)

Parameter	Unit	Moulding sand			Unit	Moulding sand		
		100 PS	50PS 50R	100R		100PS	50PS 50R	100R
Arsenic	mg/kg ds	0.018	0.102	0.019	mg/dm^3^	0.0018	0.010	0.0019
Barium	mg/kg ds	1.84	1.46	0.584	mg/dm^3^	0.184	0.146	0.0584
Cadmium	mg/kg ds	0.029	0.044	0.022	mg/dm^3^	0.0029	0.0044	0.0022
Chromium—total	mg/kg ds	<0.030	0.089	0.240	mg/dm^3^	0.0030	0.0089	0.024
Copper	mg/kg ds	0.143	0.306	0.525	mg/dm^3^	0.0143	0.0306	0.0525
Mercury	mg/kg ds	<0.005	<0.005	0.0060	mg/dm^3^	<0.0005	0.0005	0.0006
Molybdenum	mg/kg ds	<0.040	<0.040	<0.040	mg/dm^3^	<0.004	<0.004	<0.004
Nickel	mg/kg ds	0.361	0.533	0.560	mg/dm^3^	0.0361	0.053	0.056
Lead	mg/kg ds	0.150	0.604	1.23	mg/dm^3^	0.015	0.060	0.123
Antimony	mg/kg ds	<0.010	<0.010	<0.010	mg/dm^3^	<0.001	<0.001	<0.001
Selenium	mg/kg ds	<0.010	<0.010	<0.010	mg/dm^3^	<0.001	<0.001	<0.001
Zinc	mg/kg ds	4.39	5.31	3.28	mg/dm^3^	0.439	0.531	0.328
Chlorides	mg/kg ds	<20	<20	<20	mg/dm^3^	<2.0	<2	<2.0
Fluorides	mg/kg ds	<1.0	<1.0	<1.0	mg/dm^3^	<0.1	<0.1	<0.10
Sulfates	mg/kg ds	51	100	83	mg/dm^3^	5.1	10	8.3
Phenolic index	mg/kg ds	0.31	0.30	0.24	mg/dm^3^	0.031	0.030	0.024
Dissolved organic carbon (DOC)	mg/kg ds	1610	2420	3120	mg/dm^3^	161	242	312
Total dissolved solids (TDS)	mg/kg ds	3780	5920	7160	mg/dm^3^	378	592	716
pH		4.4	4.1	3.1		4.4	4.1	3.1
Total organic carbon (TOC)	mg/kg ds	7400	13,100	20,000	mg/dm^3^	740	1310	2000
Benzene, toluene, ethylbenzene, xylenes—total (BTEX)	mg/kg ds	0.19	0.70	0.60	mg/dm^3^	0.019	0.07	0.06
Polychlorinated Biphenyls—total (PCB)	mg/kg ds	<0.020	<0.020	<0.020	mg/dm^3^	<0.002	<0.002	<0.002
Mineral Oil (C10–C40)	mg/kg ds	9.17	12.1	11.3	mg/dm^3^	0.917	1.21	1.13
Polycyclic aromatic hydrocarbons—total (PAHs)	mg/kg ds	<0.10	<0.10	<0.10	mg/dm^3^	<0.01	<0.01	<0.01
Water content	%	<0.5	<0.5	<0.5	%	<0.5	<0.5	<0.5

**Table 2:** Maximum possible concentrations of leached substances and TCLP (regulatory levels) toxicity threshold values

Parameter	The requirements for waste landfill in a landfill neutral waste (basic parameters), mg/kg ds (ds—dry substance)	Maximum concentration of contaminations for toxicity characteristic US EPA TCLP criteria mg/dm^3^
Arsenic	0.5	5.0
Barium	20	100.0
Cadmium	0.04	1.0
Chromium—total	0.5	5.0
Copper	2	25
Mercury	0.01	0.2
Molybdenum	0.5	
Nickel	0.4	20
Lead	0.5	5.0
Antimony	0.06	
Selenium	0.1	1.0
Zinc	4	250
Chlorides	800	
Fluorides	10	
Sulfates	1000	
Phenolic index	1	
Dissolved organic Carbon (DOC)	500	
Total dissolved solids (TDS)	4000	
Total organic carbon (TOC)	30,000	
Benzene, toluene, ethylbenzene, xylene—total	6	benzene 0.5
Polychlorinated biphenyls— total (PCB)	1	
Mineral oil (C10–C40)	500	
Polycyclic aromatic hydrocarbons—total (PAHs)	1	
	-	

**Fig. 2:**
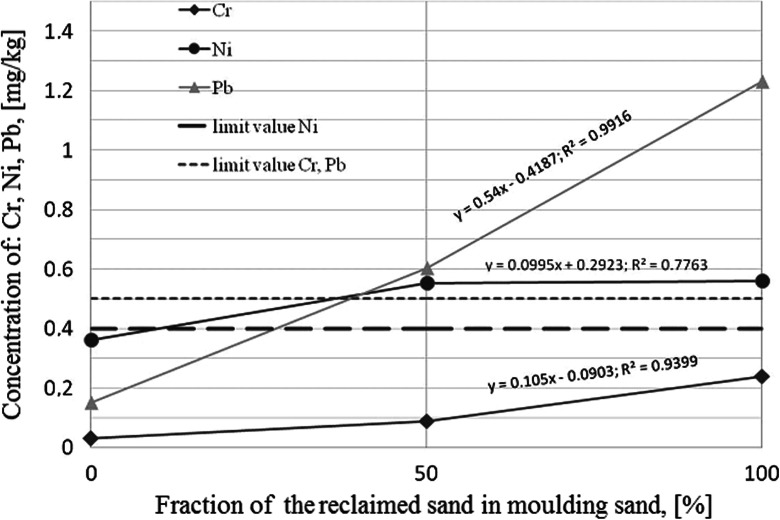
The influence of reclaimed sand addition on concentration of Cr, Ni, and Pb in leachates

Heavy metals: copper, chromium, nickel, and lead indicate a tendency of accumulating in a moulding sand as far as a fresh sand was substituted by a reclaimed sand, and thus, their concentration in filtrates was increasing when the reclaimed sand fraction in matrices was increasing (Fig. 2). The BTEX concentration in the filtrate increased three times when 50 % of fresh sand was substituted by the reclaimed sand. The applied method of the PAHs and PCB determination is characterized by too low accuracy and it is not possible to state whether a reclaimed sand addition into a moulding sand matrix influences amounts of these substances entering the solution (in case of PAHs—for all tested moulding sands—these are values <0.002 mg/dm^3^, while for PCB values <0.01 mg/dm^3^).

**Fig. 3:**
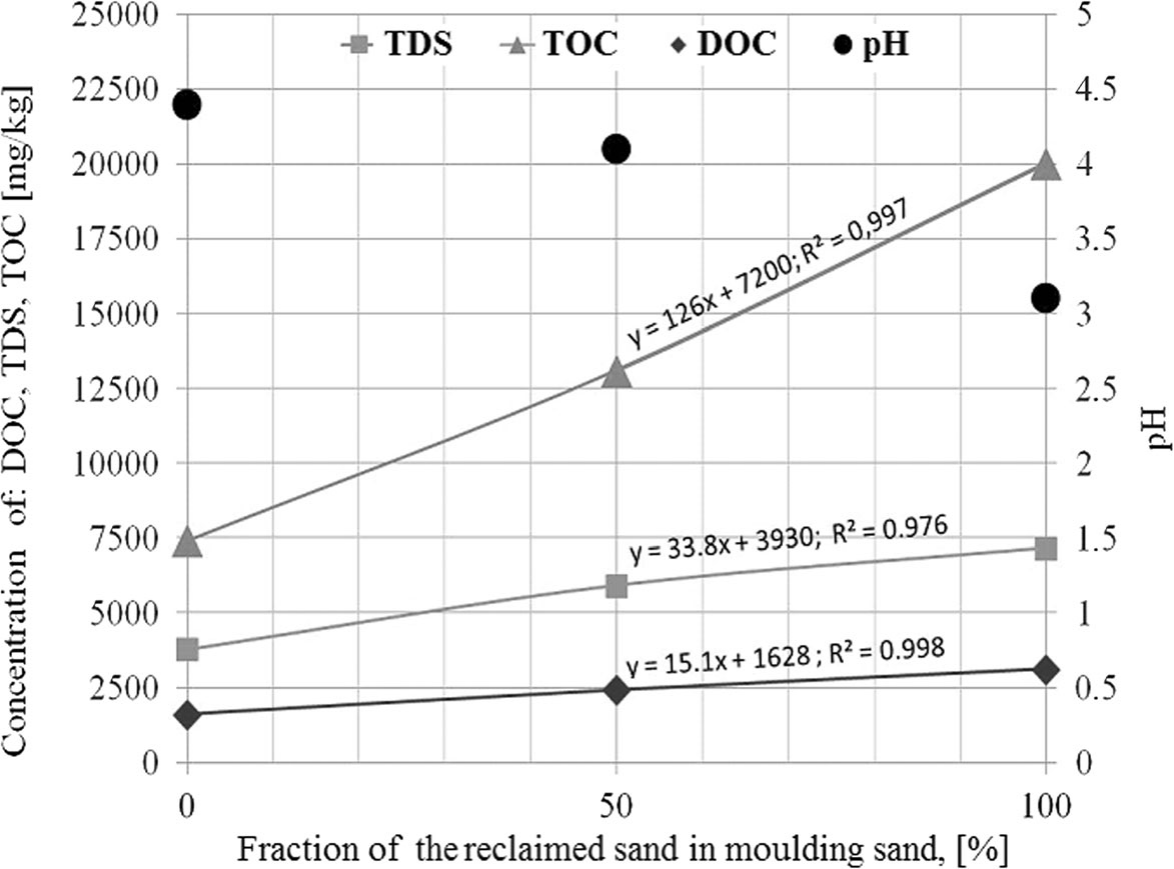
The influence of reclaimed sand addition on concentration of TOC, DOC, and TDS in leachates

Reclaimed sand additions to the moulding sand matrix causes increases of elution of substances containing organic carbon marked as: dissolved organic carbon (DOC) and total organic carbon (TOC) (Fig. 3). When the moulding sand matrix consisted in 100 % of the reclaimed sand, nearly twice as much of DOC and nearly three times as much of TOC were eluted. In addition, nearly twice as much of solid dissolved substances (TDS) were eluted. It is characteristic that at the reclaimed sand additions the pH value of the obtained filtrate was systematically lowered. Thus, for the moulding sand on the fresh sand matrix the pH value was 4.4, while for the sand on the 100 % reclaimed sand matrix pH was only 3.1. It was caused by introducing a reclaimed sand of an acidic reaction in place of high-silica sand of a neutral reaction.

Wastes of the tested moulding sands prepared on fresh sand can be stored in the dumping site of neutral wastes (zinc concentration can exceed the limiting value—given in Table 2—in case when dumping site are equipped with systems collecting effluents which are later sent to the sewage-treatment plant). The obtained filtrate contained only larger than allowable zinc and dissolved organic carbon amounts. The increased zinc content was probably caused by this element presence in cast iron poured into a mould, but its concentration could be controlled by a galvanized waste fraction in a charge. Whereas a filtrate obtained from moulding sands prepared on the matrix with a reclaimed sand addition, apart from increased concentrations of the above mentioned substances, revealed additionally higher than allowable concentrations—for neutral wastes—of lead and solid dissolved components. Filtrates obtained from all tested moulding sands were meeting requirements in force for dumping site for wastes, other than dangerous and neutral. The obtained filtrates were characterized by a relatively low pH value, nearly three. This problem can be solved by mixing, e.g., moulding sands of an alkaline reaction with the ones of an acidic reaction, if the foundry plant uses these two kinds of moulding sands. All investigated moulding sands meet the TCLP test requirements.

### Emissivity from Moulding Sands

#### Emissivity of BTEX Group

The results of gases—from the BTEX group—emissivities from moulding sands prepared on the fresh sand matrix and with a reclaimed sand addition, are given in Table 3. An increase of a reclaimed sand fraction in the moulding sand matrix causes a significant increase of the emitted gases volume. From the moulding sand on the matrix of 100 % reclaimed sand nearly twice more gases are emitted than from the moulding sand on the fresh sand matrix (14.945 dm^3^/kg sand and 24.669 dm^3^/kg sand, respectively). The process of gases emission stops after approximately 150 s from pouring liquid metal into a mould.

**Table 3:** Concentration of BTEX formed during thermal decomposition of moulding sands

Moulding sand	LOI %	Volume of gases, dm^3^/kg moulding sand	Emission of gases, mg/kg moulding sand				
			Benzene	Toluene	Ethylbenzene	Xylenes	∑BTEX
100PS	1.32	14.945	654.5	10.1	0.6	1.2	666.4 ± 66.6^*^
50PS50R	2.18	19.933	1198.6	89.0	1.8	7.7	1297.1 ± 129.7
100R	3.15	24.669	1770.4	122.1	2.2	11.8	1906.1 ± 190.6^*^

**Fig. 4:**
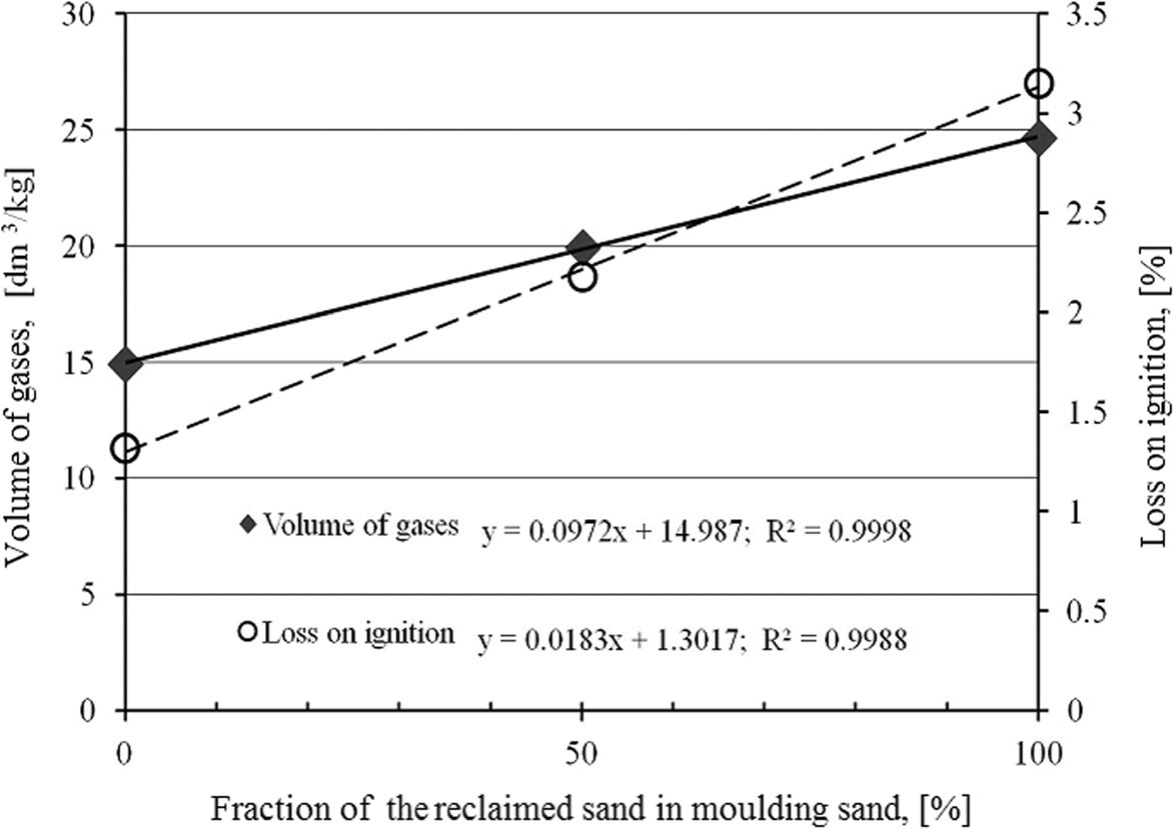
Dependence of gases volume emitted during a thermal decomposition of moulding sands and loss on ignition on the reclaimed sand fraction (recalculated to 1 kg of the moulding sand)

**Fig. 5:**
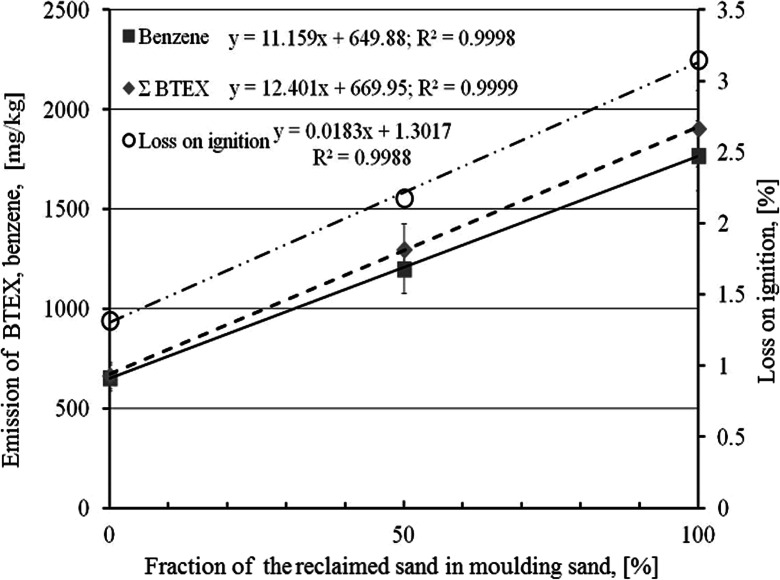
Dependence of the BTEX sum and benzene amounts formed during a thermal decomposition of moulding sands and loss on ignition on the reclaimed sand fraction (recalculated to 1 kg of the moulding sand)

The main gas from the BTEX group is benzene. Amounts of the remaining gases from this group are much smaller but these gases concentration increases with an increase of the reclaimed sand fraction in the moulding sand. The dependence of the volume of the gases and emission of compounds from the BTEX group on the reclaimed sand fraction in the moulding sand matrix in relation with losses on ignition of the given sand are presented in Figs. 4 and 5 respectively.

#### Emissivity of PAHs Group

Data concerning the PAHs amounts emitted from moulding sands of various reclaimed sand fractions, are given in Table 4. Amounts of PAHs which precipitated on a filter (together with dusts) as well as amounts that adsorbed on a polyurethane foam (in a gaseous form) were taken into account in calculations.

**Table 4:** Concentration of PAHs formed during thermal decomposition of moulding sands

Substances (No CAS)	Moulding sand/concentration, mg/kg moulding sand		
	100PS	50PS50R	100R
Naphthalene 91-20-3	5.42	10.98	8.79
Acenaphthene 83-32-9	0.00	0.00	0.00
Fluorene 88-73-7	0.18	0.94	0.46
Phenanthrene 85-01-8	0.60	1.60	2.89
Anthracene 120-12-7	0.19	0.44	0.94
Fluoranthene 206-44-0	0.81	4.72	5.97
Pyrene 129-00-0	1.97	3.03	2.84
Benz(a)anthracene 56-55-3	1.23	0.21	0.48
Chrysene 218-01-9	0.10	0.18	0.63
Benzo(b)fluoranthen 205-99-2	0.22	0.49	1.01
Benzo(k)fluoranthene 207-08-9	0.28	0.15	0.28
Benzo(a)pyrene 50-32-8	0.21	0.47	0.82
Dibenz(a,h)anthracene 53-70-3	0.22	0,00	0.00
Benzo(g,h,i)perylene 191-24-2	0.07	0.36	0.40
Indeno(1,2,3-c,d)pyrene 193-39-5	0.18	0.00	0.00
Total PAHs ± 20 %	11.76 ± 2.35	23.64 ± 4.73	25.56 ± 5.11

**Fig. 6:**
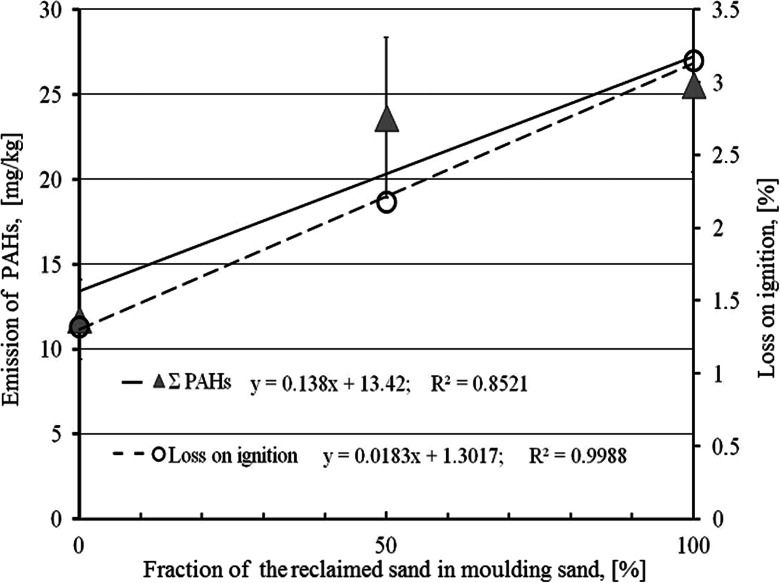
Dependence of the sum of generated substances from the PAHs group and loss on ignition on the reclaimed sand fraction in the moulding sand

The diagram of the dependence of the emitted substances from the PAHs group on the reclaimed sand fraction in the matrix is shown in Fig. 6. It can be assumed that this dependence is nearly linear, similar to changes of losses on ignition depending on the reclaimed sand fraction.

##### Moulding Sand 100PS

Naphthalene, which constituted nearly 50 % of all generated PAHs substances, was fully precipitated on the foam. Subsequent—as far as the concentration values are concerned—substances from the PAHs group are the following: pyrene, benzo(a)anthracene and fluoranthene. A concentration of benzo(a)pyrene was relatively small (0.21 mg/kg moulding sand).

##### Moulding Sand 50PS50R

Similar quantitative relations and kinds of substances from the PAHs group, which precipitated on a foam and on a filter, occur for the moulding sand prepared on the matrix consisting in 50 % of fresh sand and in 50 % of a reclaimed sand. Also in this case, the main component was naphthalene (nearly 50 % of all PAHs), which precipitated only on a foam. Subsequent substances, in respect of amounts, were fluoranthene and pyrene.

##### Moulding Sand 100R

In the moulding sand prepared on the matrix being in 100 % a reclaimed sand, naphthalene constituted app. 30 % of all substances emitted from the PAHs group. Apart from pyrene and fluoranthene, significant amounts of phenanthrene occurred.

It should be noted that along with increasing the reclaimed sand fraction in the moulding sand the concentration of benzo(a)pyrene, which attached to dusts precipitated mainly on a filter, was increasing.

## Summary and Conclusions

For the total assessment of the moulding sands harmfulness for employees and for the environment, it is necessary to perform investigations concerning the dangerous substances elution into the environment during their management and storage, as well as investigations concerning emissions of hazardous substances (especially from the BTEX and PAHs group) during moulds pouring with liquid metal, cooling, and casting knock out.

Both kinds of investigations indicated that reclaimed sand additions to moulding sands have significantly negative influence on the environment and working conditions. This concerns mainly carbon substances, which amounts are increasing along with the reclaimed sand fraction increase. A special attention should be directed to a significant increase of the emission of substances from the BTEX and PAHs group.

1. The leaching metals are very low except special elements existed as a composition of casting (Cr). Along with the reclaimed sand fraction in the moulding sand increase amounts of eluted metals: Cr, Cu, Ni, and Pb also increase. It is partially caused by lowering of the pH value of the moulding sand prepared on the reclaimed sand only (pH = 3.1) in relation to the moulding sand prepared on the fresh sand (pH = 4.4). More acidic solution favors solving of metals.
2. Carbon compounds (DOC and TOC) and TDS significantly increase their concentrations in eluents when the reclaimed sand fraction in the moulding sand increases. The DOC limits of dumping site for neutral wastes are exceeded by all tested moulding sands, while in case of TDS the requirements are only met by the moulding sand prepared on the fresh sand.
3. All tested moulding sands met the US EPA TCLP criteria (regulatory level) in the elution range.
4. An increased reclaimed sand fraction in the moulding sand caused the LOI value increase and—in consequence—the emission of larger volumes of gases. Increases of these both parameters were proportional and could be described by the rectilinear function of *R*^2^ = 0.999.
5. Along with the reclaimed sand fraction in the moulding sand increase concentrations of substances from the BTEX group, generated during mould pouring, are significantly increasing (nearly three times). The benzene (main component of this group of carcinogenic properties) concentration is also increasing nearly three times. All these concentrations are proportional to the LOI values. The BTEX sum increase is described by the equation: *y* = 12.401x + 669.95 at *R*^2^ = 0.9999, and the benzene concentration increase:
  *y* = 11.159 + 649.88 at *R*^2^ = 0.9998.
6. An abrupt change in emission of the PAHs group occurred for the moulding sand containing 50 % of the reclaimed sand in its matrix in relation to the moulding sand containing only the fresh sand (three times increase). Further reclaimed sand additions were causing only a small increase of the PAHs amount. The PAHs emission can be described by the equation: *y* = 0.138x + 13.42 at *R*^2^ = 0.8521.
7. Nearly 50 % of the substances from the PAHs group emitted in the process of the mould pouring with liquid metal constituted naphthalene, which was practically fully adsorbed on the polyurethane foam.

It should be noted that along with the reclaimed sand fraction increase, the concentration of carcinogenic benzo(a)pyrene, which attached to dusts precipitated mainly on a filter, was increasing.

## Abbreviations

DOC: dissolved organic carbon
TOC: total organic carbon
BTEX: benzene, toluene, ethylbenzene, xylenes
PCB: polychlorinated biphenyls
PAHs: polycyclic aromatic hydrocarbons
LOI: loss on ignition
WFS: waste foundry sand
HAP: hazardous air pollutant
TCLP: Toxicity Characteristics Leaching Procedure
EPA: Environmental Protection Agency
PS: silica sand
R: reclaimed sand
TDS: total dissolved solids

## Footnotes

1 Raw polyurethane foam 0.022 g/cm^2^ density (RESTEK) for collection of semi volatiles (pesticides, PCBs, PAHs).

2 Both layers of active carbon were analyzed (A and B). It was assumed that, if the amount of BTEX in the control layer (B) was lower than 10 % of these substances concentration in the main adsorption layer (A), none ‘breakthrough’ occurred and the measurement was the proper one.
